# Tangled up in two: a burst of genome duplications at the end of the Cretaceous and the consequences for plant evolution

**DOI:** 10.1098/rstb.2013.0353

**Published:** 2014-08-05

**Authors:** Kevin Vanneste, Steven Maere, Yves Van de Peer

**Affiliations:** 1 Department of Plant Systems Biology, VIB, 9052 Ghent, Belgium; 2 Department of Plant Biotechnology and Bioinformatics, Ghent University, 9052 Ghent, Belgium; 3 Genomics Research Institute (GRI), University of Pretoria, 0028 Pretoria, South Africa

**Keywords:** whole genome duplication, K–Pg boundary, extinction event, innovation, speciation, plant evolution

## Abstract

Genome sequencing has demonstrated that besides frequent small-scale duplications, large-scale duplication events such as whole genome duplications (WGDs) are found on many branches of the evolutionary tree of life. Especially in the plant lineage, there is evidence for recurrent WGDs, and the ancestor of all angiosperms was in fact most likely a polyploid species. The number of WGDs found in sequenced plant genomes allows us to investigate questions about the roles of WGDs that were hitherto impossible to address. An intriguing observation is that many plant WGDs seem associated with periods of increased environmental stress and/or fluctuations, a trend that is evident for both present-day polyploids and palaeopolyploids formed around the Cretaceous–Palaeogene (K–Pg) extinction at 66 Ma. Here, we revisit the WGDs in plants that mark the K–Pg boundary, and discuss some specific examples of biological innovations and/or diversifications that may be linked to these WGDs. We review evidence for the processes that could have contributed to increased polyploid establishment at the K–Pg boundary, and discuss the implications on subsequent plant evolution in the Cenozoic.

## 1. Introduction

Flowering plants typically have large genome sizes and contain many genes, the majority of which evolved during the past 250–300 Myr through gene duplication [1]. A particularly striking feature of plant genomes, also explaining their large sizes, is the large number of whole genome duplications (WGDs) that have been uncovered [2–4]. It is now commonly accepted that one WGD occurred in the ancestor of all seed plants, and an extra one in the ancestor of all flowering plants, so that every extant angiosperm is in fact a palaeopolyploid containing the remnants of at least two WGDs [5]. Furthermore, a hexaploidy event pre-dates the origin of all core eudicots, which make up approximately 75% of extant angiosperm diversity [6–8], whereas traces of a WGD at the base of the monocots also suggest a WGD shared by most, if not all, monocots [9]. In addition, several more recent independent WGDs have been unveiled in many different plant lineages. As a result, the genomes of some extant plant species carry the remains of up to six successive genome duplications [10]. Here, we focus on the more ‘recent’ palaeopolyploidizations that occurred in the past 100 Myr, a large fraction of which seemingly took place around the Cretaceous–Palaeogene (K–Pg) extinction event at 66 Ma [11]. We have an in-depth look at this wave of WGDs associated with the K–Pg boundary, many of which pre-date lineage diversifications that resulted in some of the largest and arguably most successful present-day plant families, often characterized by particular biological innovations. Finally, we review processes that can explain these observations, and discuss how these palaeopolyploidizations could have influenced plant evolution in the Cenozoic.

## 2. A burst of genome duplications at the Cretaceous–Palaeogene boundary

In 2009, we described a tentative link between many of the known palaeopolyploidization events in plants and the K–Pg boundary, and speculated that WGD was linked to plant survival around that time [11]. Although many found this an interesting hypothesis [12], most remained sceptical, in particular because of the limited amount of data available at that time, and because dating ancient events that occurred tens of millions of years ago is often problematic. Only six complete genome sequences and a few transcriptome assemblies were available for analysis in 2009, limiting both the taxon sampling and possibility to implement proper primary fossil calibrations. Dating was performed using a penalized-likelihood inference method that incorporates an autocorrelated relaxed clock model, which assumes that branches that share a direct common ancestor also share similar evolutionary rates [13]. This assumption seems unlikely however, in the light of the sparse taxon sampling considered [14], and violation thereof may lead to inconsistent age estimates [15]. Calibrations were typically implemented as fixed secondary point calibrations, which may lead to illusionary precision of the time estimates [16].

Recent years have seen a huge increase in plant (whole genome) sequence data [17], in addition to the development of more powerful Bayesian methods for sequence divergence estimation [18–20], as well as more powerful high-performance computing systems that allow such intensive Bayesian algorithms to be run on a massive scale. We therefore recently revisited the hypothesized link between the K–Pg mass extinction and successful WGDs [21]. We used plant genome sequence information from a total of 41 species representing a broad coverage of the overall angiosperm phylogeny, incorporating 38 full genome sequences and three transcriptome assemblies, greatly improving taxon sampling with respect to the previous study [11]. In total, 20 independent WGDs could be dated compared with nine previously by dating all their identifiable homeologues created by the WGD event. For WGDs for which genome sequence information was available for several descendant species (e.g. WGDs preceding the divergence of Solanaceae, Fabaceae or Poaceae—see further), this WGD was dated independently for each species to assess their individual age estimates. Absolute age distributions were then constructed for each species WGD, for which a consensus WGD age estimate was obtained by taking the mode of its kernel density estimate, which is more flexible in comparison with traditional parametric distributions because it allows a better exploration of the true underlying shape of the distribution [22], whereas 90% confidence intervals were obtained through a bootstrapping procedure [23]. Dating itself was carried out with the Beast package [20], using an uncorrelated relaxed clock model that assumes a lognormal distribution on evolutionary rates [19], and therefore should be better equipped to deal with rate shifts between different branches compared with autocorrelated relaxed clocks when taxon sampling is limited [24]. Proper calibration priors in Bayesian time estimation are of paramount importance as they can have a profound impact on the posterior age estimates [15,25–28]. Primary fossil calibrations were implemented as flexible lognormal calibration priors that represent the error associated with the age of the fossil in a more intuitive manner [27,29]. Fossils have a hard minimum bound corresponding to the earliest age to which the fossil can reliably be attributed to. The peak mass probability can be put at some distance after this earliest age to accommodate for the lag between first fossil occurrence and the actual divergence event the fossil is used to describe. Lastly, the lognormal distribution has an infinite extending, but small probability tail that can be used as a soft maximum bound to account for the uncertainty associated with choosing proper maximum bounds for fossil calibrations. More detailed information can be found in Vanneste *et al.* [21].

**Figure 1.**
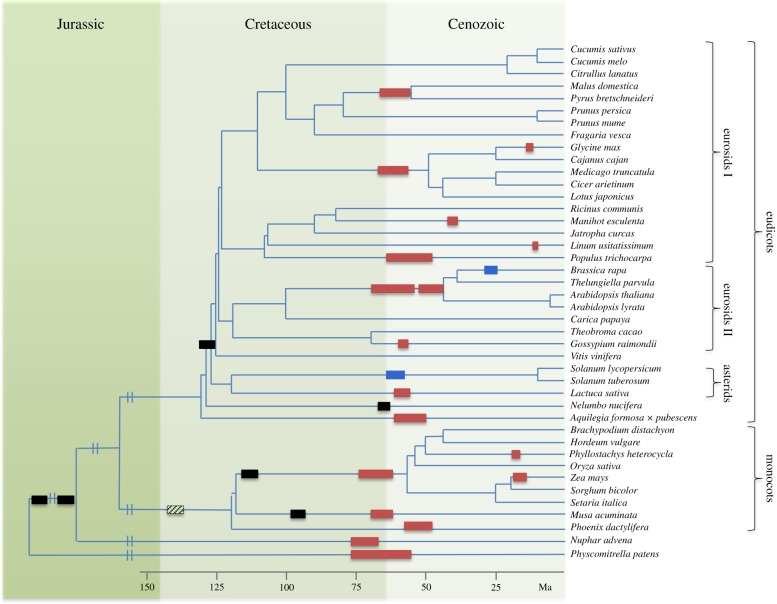
A wave of WGDs is associated with the K–Pg boundary approximately 66 Ma. The figure illustrates the tree topology for the green plants with all known WGDs indicated by bars. Red and blue bars represent 90% confidence intervals on dated tetraploidies and hexaploidies, respectively. Black bars represent WGD age estimates from literature [21]. A possible WGD at the base of the monocots is indicated by a dashed bar, because its exact phylogenetic placement remains unclear [9]. The WGD for *Populus trichocarpa* and the one shared by *M. domestica* and *P. bretschneideri* are corrected WGD age estimates based on fossil minimum boundaries and/or other dating studies [21]. Branch lengths are truncated after 150 Ma to allow a better overview. Figure adapted from Vanneste *et al.* [21].

An updated overview of palaeopolyploidizations is summarized in figure 1 [21]. Although dating of such ancient events surely remains a challenging exercise, and WGD dates are subjected to change as more plant sequence data and powerful dating methods become available [12,30,31], many plant palaeopolyploidizations were again found to cluster at the K–Pg boundary [21], supporting our previous observations [11].

## 3. Implications of genome duplications associated with the Cretaceous–Palaeogene boundary

The increased long-term survival of WGDs around the K–Pg boundary appears indicative of enhanced polyploid plant establishment at that time, either because WGDs provided a selective advantage for polyploids compared with their diploid progenitors, or alternatively, because the cataclysmic events that took place 66 Ma were responsible for the production of an excess of polyploids (see further). However, whether cause or effect, many of these WGDs pre-date the radiation of some very large and successful plant families with particular biological innovations. Similar observations can be done in other parts of the tree of life, where WGDs are often found at branches leading to species-rich clades, such as more than 25 000 species of teleost fishes and more than 350 000 species of flowering plants [3,32]. On the other hand, one should be cautious not to over-interpret the importance of WGDs for species radiations. For instance, in vertebrates, it was suggested that the often-quoted correlation between the teleost fish WGD and increased post-WGD diversity and/or complexity does not hold when extinct basal lineages were considered [33]. Teleost fish evolution rather fits a more nuanced pattern of reduced extinction risk after WGD, resulting in a lag period between WGD and its effect on species diversity and/or complexity [34]. Additionally, it was recently demonstrated that an extended period of about 40–50 Myr passed between the salmonid-specific WGD and strong lineage diversification, suggesting the latter was probably mostly driven by climatic factors [35]. Below, we first examine a few examples of biological innovations (or better said, elaborations thereof [2]) that can reliably be traced back to WGDs located at the K–Pg boundary in plants, focusing on fleshy fruits in the Solanaceae and advanced nodulation characteristics in the papilionoids, before taking a deeper look at evidence whether or not these WGDs could have directly enhanced speciation.

### (a) Biological novelty

#### (i) Fleshy fruits

**Figure 2.**
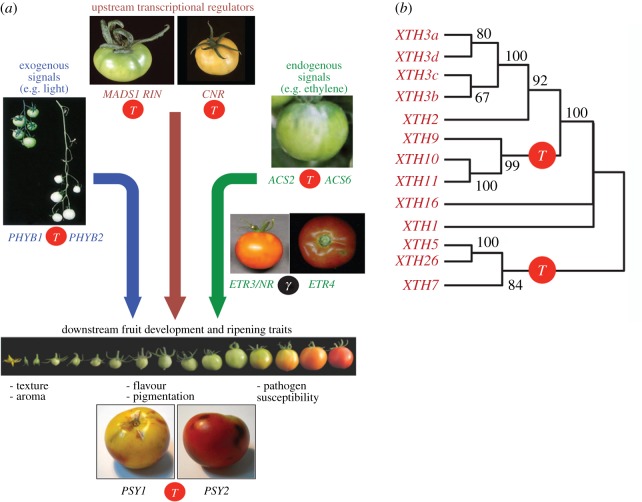
The Solanaceae-specific genome triplication contributed to the evolution of the tomato fruit. (*a*) Illustration of the fruit-ripening control network. The upstream transcriptional regulators *MADS-RIN* and *CNR*, in combination with the enzyme ACC synthase (*ACS*), control the production of the ripening hormone ethylene. Ethylene receptors (*ETR*) drive expression changes in several output genes, including phytoene synthase (*PSY*), which is the rate-limiting step in carotenoid biosynthesis. Light influences fruit pigmentation through an ethylene-independent pathway mediated by phytochromes (*PHY*). Several key component paralogous gene pairs (*MADS1*/*RIN*, *PHYB1*/*PHYB2*, *ACS2*/*ACS6*, *PSY1*/*PSY2*) were generated by the genome triplication (*T*, red circle), whereas *ETR3*/*ETR4* was created by the core eudicot shared hexaploidy (*γ*, black circle). (*b*) Illustration of the expansion by both genome triplication (*T*, red circle) and tandem duplications of a family of xyloglucan endotransglucosylase/hydrolases (*XTHs*), which control fruit-ripening through modification of cell wall structure and composition. Figure adapted from Sato *et al.* [38].

The fleshy fruits observed in some plant lineages are an important biological innovation that serves to enhance seed distribution by attracting vertebrate frugivores for long-distance seed dispersal, and hence increases plant success [36]. Specialization of the fleshy fruit for particular (groups of) vertebrates may also enhance speciation [37]. Based on the recently published genome of tomato (*Solanum lycopersicum*), a genome triplication event in the Solanaceae shared with potato (*Solanum tuberosum*) was firmly established [38] and dated at the K–Pg boundary (figure 1). Many new gene family members with important fruit-specific functions were created through this WGD. Figure 2*a* illustrates several genes in the fruit-ripening control network that are paralogues with different physiological roles generated through the genome triplication. These include, for instance, the transcription factors and enzymes necessary for ethylene biosynthesis (*MADS1/RIN*, *CNR* and *ACS2*/*ACS6*), red light photoreceptors influencing fruit quality (*PHYB1*/*PHYB2*), and also some effector genes mediating lycopene biosynthesis (*PSY1*/*PSY2*) that control fruit pigmentation. Endogenous ethylene receptors (*ETR3*/*ETR4*) created by the eudicot-wide genome duplication also participate in this network. Similarly, fruit texture is controlled in part by over 50 genes that encode proteins involved in modification of cell wall structure and composition, and show differential expression during fruit development and ripening. Figure 2*b*, for instance, illustrates the expansion, through genome triplication and subsequent tandem duplications, of a family of xyloglucan endotransglucosylase/hydrolases (*XTHs*) involved in determining fruit texture. Differential loss between tomato and potato of one of the triplicated members, *XTH10*, suggests that genetic specialization, and hence diversification between the different members of the Solanaceae, was facilitated by the triplication event [38]. It should however be noted that fleshy fruits exist in many different plant lineages, many of which are not marked by a specific polyploidy, emphasizing that the Solanaceae-shared WGD contributed several genes that were later incorporated into more elaborate fleshy fruit development, so that the latter represents an ‘elaboration’ rather than a true ‘innovation’ [2].

#### (ii) Rhizobial nodulation

**Figure 3.**
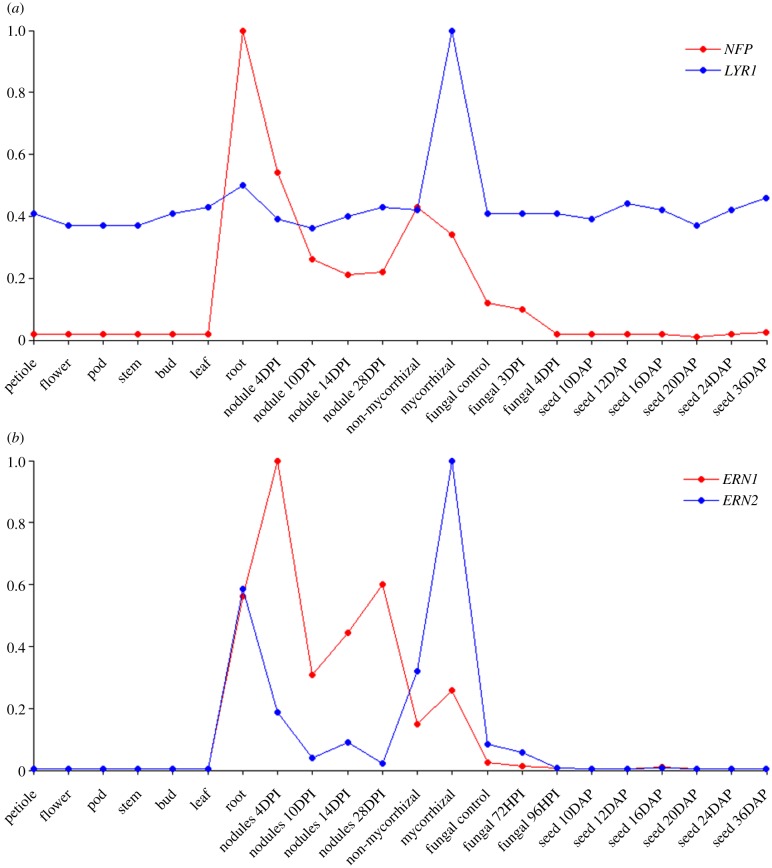
The papilionoid genome duplication contributed to the evolution of nodulation. Paralogues created by WGD, (*a*) *NFP* and *LYR1*, and (*b*) *ERN1* and *ERN2*, display contrasting expression patterns, suggesting functional specialization. *NFP* and *ERN1* are expressed predominantly in the nodule, whereas *LYR1* and *ERN2* are highly expressed during mycorrhizal colonization. The average transcript levels of three replicates are shown, scaled by dividing each data point by the maximum mean transcript level across all experiments. HPI, hours past inoculation; DPI, days past inoculation; DAP, days after pollination. Figure adapted from Young *et al.* [41].

A common feature of most papilionoid legumes is rhizobial nodulation, the formation of specialized organs called root nodules, which host nitrogen-fixing rhizobial symbionts. Nodulation is a biological innovation that allows growth on nitrogen-deprived soils, because plants receive fixed nitrogen from their symbionts, in return for a steady supply of carbon and energy sources [39]. Specialization for different rhizobial symbionts may also have aided papilionoid speciation [40]. Analysis of the genome sequence of *Medicago truncatula* confirmed that the papilionoid-shared WGD, also located at the K–Pg boundary (figure 1), has played an important role in the evolution and elaboration of rhizobial nodulation [41]. Nodulation is initiated when the plant signalling system comes into contact with specific bacterial Nod factors, which in papilionoids evolved a distinctly nodulation-specific function [42]. Analysis of the *M. truncatula* genome showed that both the Nod factor receptor *NFP* and transcription factor *ERN1* have paralogues, *LYR1* and *ERN2*, respectively, which originated through the papilionoid WGD. Figure 3 illustrates that both gene pairs show divergent expression patterns, reflecting functional specialization. *NFP* and *ERN1* are expressed predominantly in the nodule and are known to be active in nodulation [43], whereas *LYR1* and *ERN2* are highly expressed during mycorrhizal colonization. This suggests that these nodulation-specific signalling components are derived from more ancient genes originally functional in mycorrhizal signalling that evolved new transcriptional functionality after the papilionoid WGD [41]. Additional support for this conclusion comes from the observation that the orthologue of *NFP* in a nodulating non-legume outgroup, *Parasponia andersonii*, functions both in nodulation and mycorrhizal signalling [44]. Interestingly, a nodulating legume outgroup that did not share the papilionoid WGD, *Chamaecrista fasciculata*, exhibits ancestral nodule characteristics in comparison with most nodulating papilionoids [45]. *Parasponia* diverged somewhere between 100 and 120 Ma from the papilionoids [46], whereas *Chamaecrista* diverged approximately 60 Ma from the papilionoids [45]. Independent of whether their last common ancestor could already perform nodulation or whether this trait evolved independently in both lineages, this would suggest that the ability for advanced nodulation characteristics was not able to evolve for about 40–60 Ma, whereas it did so very rapidly after the papilionoid WGD [45]. This emphasizes that although the papilionoid WGD was not an absolute prerequisite for the evolution of nitrogen-fixing nodulation, it most likely facilitated the development of several elaborate papilionoid nodule forms.

To assess the contribution of the papilionoid WGD to *M. truncatula* nodulation in more detail, Young *et al.* [41] also investigated the expression of 618 homeologous gene pairs from six different organs based on RNA-seq data for one or both homeologues, to determine the number of genes showing organ-enhanced expression (defined as having expression in a single organ that is at least twice the level in any other). A large fraction of homeologues demonstrated organ-specific enhanced expression. Among homeologous gene pairs with nodule-enhanced expression, a single paralogue was nodule-enhanced in 43 of 51 gene pairs, with the other eight gene pairs showing nodule-enhanced expression for both gene pairs. Of 142 transcription factors derived from the papilionoid WGD for which RNA-seq data was available, 11 showed such enhanced nodule expression. These results indicate that many homeologous genes, in particular signalling components and regulators, were retained after the papilionoid WGD and gained specialized roles in nodulation afterwards. However, some other nodule-related genes were found to derive from the core-eudicot-specific hexaploidy. This confirms a more complex model wherein the capacity for primitive interaction with new symbionts evolved quite early, derived from the existing mycorrhizal machinery, explaining the evolution of nodulation in multiple plant lineages [39,47], after which the papilionoid WGD allowed the creation of additional genes that were incorporated into the development of more advanced nodulation characteristics [41]. A recent integrated comparative genomic approach based on the sequenced genomes of four papilionoid species (*M. truncatula*, *Lotus japonicus*, *Glycine max* and *Cajanus cajan*) supports this by demonstrating that many of the approximately 25% of WGD-derived duplicate pairs that have been retained, show high levels of expression divergence and function in different processes required for successful nodulation [48].

### (b) Speciation

The previous examples of biological innovations originating through the retention of WGD duplicates suggest that WGDs, through assisting biological innovations and diversifications, might also facilitate speciation. For instance, as stated previously, specialization for interactions between particular vertebrate frugivores for seed dispersal in fleshy fruits or with specific rhizobial symbionts in nodulation, might aid speciation. However, the question remains whether WGD itself can also actively promote speciation. Some of the WGDs associated with the K–Pg boundary (figure 1) pre-date extremely successful plant lineages characterized by species radiations following the WGD event. These include the Brassicaceae (approx. 3700 species), Poaceae (approx. 10 000 species), Asteraceae (approx. 23 600 species), Solanaceae (approx. 2460 species) and Fabaceae (approx. 19 500 species). Many of these, however, have a species-poor sister group that shared the WGD event, which led to the development of the WGD-radiation lag time model that emphasizes that the success of these plant families should be viewed in the light of their specific evolutionary routes taken [49]. Even the limited set of species in figure 1 demonstrates that many present-day plant families, such as the Cucurbitaceae, represented by *Cucumis melo*, *Cucumis sativus* and *Citrullus lanatus*, did not undergo any WGD in the past approximately 100 Myr. Using the number of species as a simple, albeit admittedly crude, measure for success, this family of about 950–980 species can also be considered fairly successful [50]. Alternatively, some plant families with a palaeopolyploid history, such as the Nymphaeaceae, have arguably not been very successful in terms of species radiation, counting only around 70 species [51]. Such observations emphasize the importance of ecological opportunity for realizing plant evolutionary potential, irrespective of polyploidization [2,49,52,53].

Nevertheless, the success of many plant families that have undergone a WGD suggests that their strong diversification may be ascribed, at least partly, to their polyploid ancestry. In an attempt to gauge the effect of WGD on speciation, Soltis *et al.* [3] tested whether such post-WGD clades displayed higher diversification rates, while accounting for the confounding effects of extinction. Although the results were considered preliminary, owing to the lack of reliable genomic data for palaeopolyploidy in combination with insufficient taxon sampling to place WGDs confidently on plant family phylogenies, a highly statistically significant relationship between diversification and WGD was found for four of the five aforementioned successful plant families. The fifth plant family, the Asteraceae, was not considered, and a statistical relationship hence remains untested. It should however be noted that the latter constitutes the single largest present-day angiosperm family [54].

The molecular mechanisms that might promote speciation after WGD are still not very well understood. One often-quoted mechanism is reciprocal gene loss (RGL), the genetic isolation of separated populations through loss of different gene copies that lead to incompatibilities when the populations encounter each other again [55,56]. Through WGD, a very large pool of loci becomes available simultaneously for divergent resolution between subpopulations, which could quickly result in reproductive isolation if essential genes are involved. Scannell *et al.* [57] demonstrated that the pattern of duplicate gene pair loss differs at 20% of all loci between three different yeast species that shared a WGD. Similarly, about 8% of ancestral *Tetraodon* and zebrafish loci were subjected to RGL after the teleost fish WGD [58]. For plants, the situation is less clear. Schnable *et al.* [59] separated the two subgenomes of modern grasses derived from the WGD shared by the Poaceae. In contrast to the aforementioned studies in yeast and teleost fishes, strong evidence of RGL between homeologues of the different subgenomes was lacking, suggesting post-WGD RGL was unlikely to be a driving force in the radiation of the grasses, although systematic studies about RGL in plants are still missing.

However, genes do not necessarily need to get lost or silenced, as other neutral scenarios after gene duplication might also promote speciation. Many genes perform multiple functions through differential expression at different developmental stages and/or tissues. Duplication of such genes often leads to subfunctionalization, the division of the subfunctions over the two daughter copies [55,60]. Alternatively, genes can have trace activity for a second function whose optimization is constrained by adaptive conflicts with the primary function, which can be resolved by optimizing the functions separately in different paralogues after duplication [61]. Reproductive isolation of such a population, for instance driven by geological phenomena that lead to geographical barriers, could lead to orthologues of the two isolated populations acquiring different subfunctions. Although F_1_ hybrids in contact zones from the two populations would develop correctly because each (sub)function is performed by one of the genes from each population, one-eighth of the F_2_ zygotes will lack one of the (sub)functions, which could be lethal if such functions are essential [62,63]. As for RGL, this effect would be exacerbated in the case of WGD, which generates a much larger number of duplicate loci that can be divergently subfunctionalized [2]. Lineage-specific subfunctionalization could therefore in theory accelerate speciation, but this remains untested.

## 4. Both neutral and adaptive processes most likely contribute towards enhanced polyploid establishment under stressful conditions

Above, we discussed new evidence that seems to provide further support for the association between plant palaeopolyploidizations and the K–Pg boundary, some of which can be linked to particularly successful biological innovations and increased diversification rates. The K–Pg boundary is especially known for its associated extinction event, which constitutes the last of the five major mass extinctions in the Phanerozoic eon [64]. This cataclysmic event most likely resulted from the combination of several factors such as increased volcanism, greenhouse warming, and in particular the bolide impact near Chicxulub (Mexico) [65], resulting in a challenging unstable environment impairing the survival of most living organisms [66]. The question remains, at a time when an estimated 75% of all species went extinct [67], why did many of the plant species we are all so familiar with probably undergo a WGD? Similar observations have been made for present-day polyploids, which are often encountered in unstable and stressful environments [68]. For instance, there is an overabundance of recently formed polyploids in the Arctic [69]. Below, we discuss two, not mutually exclusive, processes that could help explain this pattern and the implications thereof for plant evolution.

### (a) The adaptive scenario

The adaptive scenario explaining polyploid success has been explored extensively in the past decade [2,3,70–73], and will therefore only be covered concisely here. This scenario is mostly based on a characteristic often displayed by newly formed polyploids, namely transgressive segregation, i.e. the formation of more extreme phenotypes in the resulting hybrid populations compared with their diploid parents [70]. This becomes more pronounced as the two parental genomes contributing to the polyploid become more diverged, especially so in allopolyploids that result from the merger of two different species, which may display strong hybrid vigour (heterosis) by virtue of possessing novel allelic combinations not found in either parent [74]. However, the exact molecular mechanisms behind hybrid vigour are still largely unknown [75], although it has been suggested recently that cells might distinguish between parental alleles based on their relative protein and mRNA stability, which therefore conserves energy otherwise required for removal of such unstable products that can be used to promote growth and expression of new favourable traits [76].

Irrespective of the exact molecular mechanisms, genomic instability and gene expression changes soon after polyploid formation may result in increased phenotypic variability of the polyploids with respect to their diploid progenitors [2]. Genomic instability refers to the extensive structural changes of the chromosomal DNA that typically take place in the first few generations after polyploidization, such as fusions, fissions, duplications, inversions, translocations and eliminations [77], often coupled to mitotic and meiotic abnormalities [78,79]. Gene expression typically changes markedly [80], in conjunction with widespread epigenetic repatterning [81], in the first few generations after polyploidization. These structural and expression changes have collectively been described as genomic shock, and in the case of allopolyploids seem to be attributable to both the hybridization process [82] and the genome doubling itself, with the latter possibly having a calming effect [83]. Although these extensive changes often result in decreased polyploid fitness and increased offspring sterility, in the light of increased phenotypic variability, they can also confer plasticity to the polyploid genome to allow quick adaptation to new environments and changing conditions [70,71,73,84,85].

Other potential adaptive advantages of newly formed polyploids include the masking of deleterious recessive alleles leading to increased genetic redundancy [86], network redundancy on a larger scale [87] and possibly even an increased capacity for phenotypic plasticity itself [88,89]. Polyploids also often exhibit traits that promote their establishment through mitigating the minority cytotype disadvantage, which is a strong negative frequency-dependent selection on the polyploid through a large proportion of ineffective matings with the diploid progenitor majority cytotype [90]. Such traits include the loss of self-incompatibility, which enables selfing, and the gain of apomixis, which enables asexual reproduction. Polyploidization is also sometimes associated with a shift from annual to perennial habit, which opens up a longer time window for successful mating. Lastly, their fast morphological and/or physiological differentiation can enhance the number of successful matings through sympatric niche separation from the diploid progenitor population [73,91,92].

### (b) The neutral scenario

A series of recent findings point to the possibility of a more neutral scenario to explain the apparent association between palaeopolyploidizations and the K–Pg boundary [21]. It has been acknowledged for a long time that the formation of unreduced gametes is the main mode of polyploid formation in plants, but the low estimates of unreduced gamete production in natural populations typically seemed too restrictive for the establishment of polyploids [93,94]. Although the chance of two unreduced 2*n* gametes meeting is very low, tetraploid occurrence is most likely facilitated by a triploid bridge, the creation of an intermediate triploid stage through the combination of an unreduced 2*n* and reduced *n* gamete [95]. Such triploids often display large fertility and fitness defects; however, they also produce enhanced levels of unreduced 3*n* gametes that can form tetraploids through backcrosses with reduced *n* gametes from the diploid progenitor population, and hence alleviate the minority cytotype disadvantage [96,97]. Accordingly, a recent general gametic modelling approach for diploid–polyploid systems that predicts equilibrium ploidy frequencies based on empirical estimates of unreduced gamete formation, demonstrated that these low levels can be adequate to explain a drift towards higher ploidy [98].

Another well-documented observation is that levels of unreduced gamete formation can be increased by external stimuli such as stress and a fluctuating environment [94,99–104]. Temperature in particular has a pronounced effect on unreduced gamete formation. Increasing temperatures to extreme levels in *Rosa* species resulted in more unreduced gametes being produced through alterations in spindle formation during meiosis II [105]. Similarly, inducing cold stress increased unreduced gamete formation in *A. thaliana* through alterations in post-meiotic cell plate formation and cell wall establishment [106]. Although hybridization itself typically also increases the levels of unreduced gamete formation in plants [107], temperature levels can potentially also enhance this hybrid trait, as witnessed in some *Brassica* interspecific hybrids after cold treatment [97]. Moreover, it became clear recently that the effect of the environment on unreduced gamete formation is most likely not limited to present-day plants. Increased levels of fossil unreduced pollen were observed in the now extinct conifer family Cheirolepidiaceae at the Triassic–Jurassic transition, which corresponds to the fourth of the five major extinction events [108]. Abnormal gymnosperm pollen [109] and lycophyte spores [110] have also been reported during the Permian–Triassic transition, corresponding to the third of the five major extinction events.

Increased unreduced gamete production during times of environmental stress and/or fluctuation could thus be an important factor in explaining the apparent clustering of palaeopolyploidizations at the K–Pg boundary [21]. It could also explain why many present-day polyploids often are more abundant in stressful environments, such as the Arctic [69] or habitats created by anthropogenic disturbance [111]. For both the K–Pg boundary and present-day examples, the association between increased polyploid establishment and environmental stress and/or fluctuation would not require any explicit adaptive advantage, but could be explained by a neutral mechanism [99] such as increased unreduced gamete formation. This is in agreement with modelling approaches that predict increased replacement of diploids by polyploids under a changing environment, without assuming any *a priori* adaptive advantage of the polyploids [112]. The effect of increased unreduced gamete production during environmental stress and/or fluctuation is even expected to be intensified through higher background extinction levels of the diploid populations [34], increasing the overall relative frequency of unreduced gametes to the total gamete pool, which would enhance the chance of successful unreduced gamete matings.

**Figure 4.**
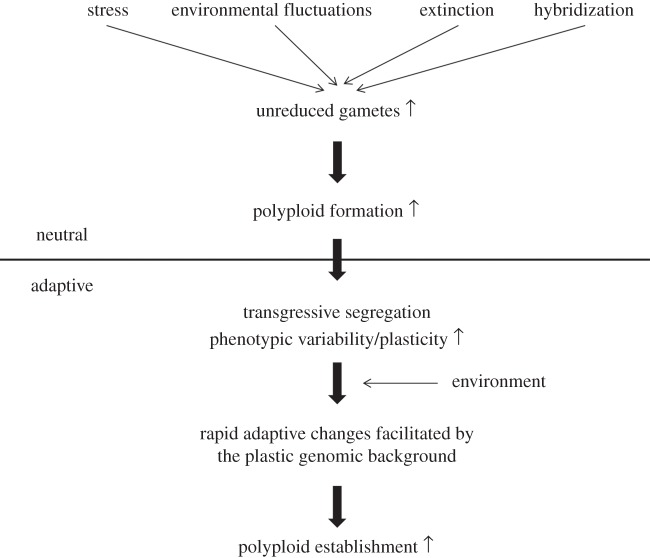
Both neutral and adaptive processes probably contribute to enhanced polyploid establishment under environmental stress and/or fluctuations. The latter likely increase the formation of unreduced gametes, whereas other processes such as hybridization and extinction of the background diploid population can also contribute to an overall increase of unreduced gametes in the total gamete pool. This is expected to lead to more polyploids being formed even in the absence of any active adaptive advantage. Transgressive segregation and genomic instability of polyploids on the other hand may lead to heterotic phenotypes, increased phenotypic variability and plasticity that, if beneficial under the changing environment, can be rapidly selected for, which is expected to lead to more polyploids being established even in the absence of increased polyploid formation. Note that irrespective of which scenario carries more weight, the environment plays an important role in polyploid establishment.

Accumulating evidence for a more prominent role of the neutral scenario does not however preclude a role for the adaptive one. Figure 4 summarizes an intertwined situation wherein environmental stress and/or fluctuation drive polyploid formation through increased unreduced gamete production, after which adaptive processes act to ensure polyploid establishment. Dependent upon specific circumstances, either the neutral or adaptive component could carry more weight. The apparent association of palaeopolyploidizations with the K–Pg boundary [21], and present-day polyploids with stressful habitats [69,111], in combination with evidence that unreduced gamete formation is a major route towards polyploidization [98] that may be intensified through environmental stress and/or fluctuations as witnessed at several large-scale extinction events [108], hints at a strong role for the neutral component. There are however many observations that also argue in favour of the adaptive component [73]. Although one has to remain cautious with generalizations about the distribution and prevalence of recent polyploids, because many exceptions can be found [113], some trends are apparent. For instance, recent polyploids appear to have larger habitat distributions, suggesting they can tolerate a wider range of ecological conditions [114–116]. Most strikingly, they are less likely to be endangered and more likely to be invasive on a worldwide scale compared with diploids [117]. Such observations would be difficult to explain purely through neutral mechanisms.

The genetic component of unreduced gamete production merits some more attention. Traditional breeding studies established that diploid gamete production is a highly heritable trait that can be enhanced in as few as two to three cycles of recurrent selection in species such as alfalfa [118] and red clover [119]. In *Arabidopsis*, a surprisingly strong tolerance of gametes to both trisomy and several other complex karyotypes exists [120], whereas several genetic players that can influence unreduced gamete production through their effect on the orientation of the spindle apparatus in male meiosis have recently been identified [121], such as *AFH14* [122], *JAS* [123] and *AtPS1* [124]. Stress-induced altered functionality of these genetic components may explain the effect of the environment on unreduced gamete production [104]. These observations open up the possibility that polyploidization might even constitute an inducible evolutionary mechanism by which plants cope with ecological disasters, much akin to the stress-inducible mutator systems such as the SOS response in bacteria [125]. The latter is a transient response to stress and changing environments by means of a set of ‘evolution genes’ that decrease replication fidelity and increase mutation rates to generate genetic diversity upon which natural selection can act [126,127]. Such evolution genes are thought to undergo biological evolution themselves through indirect selection, and their presence in higher organisms has been hypothesized [128]. Because all extant angiosperms shared at least two rounds of WGD [5], with an extra shared WGD at the base of the core eudicots [6] and possibly also the monocots [9], recurring WGD events [2–4] could have maintained residual heritable genetic variation in diploid plants for the ability to produce unreduced gametes and form polyploids in times of ecological upheaval. Despite a genetic component, this does not need to be necessarily under the direct control of any adaptive programme, as it could just as well primarily be an ‘evolutionary spandrel’ that received secondary functionality [129]. In any case, such a system could provide an alternative for the mutator systems in bacteria, which would be less efficient in plants owing to their smaller effective population sizes and longer life cycles, but this remains currently entirely hypothetical.

## 5. Enhanced establishment of polyploids at the Cretaceous–Palaeogene boundary may have paved the way for angiosperm success in the Cenozoic

The neutral and adaptive processes described above offer a framework for the apparent clustering of WGDs at the K–Pg boundary, but fail to explain their long-term success in terms of speciation and biological novelty. For all examples we considered, it was apparent that the duplication of the whole genome provided an increase in raw genetic material on which evolution could work. In accordance with Ohno's classical models [130,131], the newly created gene copies could undergo neofunctionalization (the creation of a new function), subfunctionalization (the division of an ancestral function or functions over the daughter copies), or be kept for dosage amplification (the production of more of a beneficial gene product) or any combination thereof as explained by more complex population genetic models [132]. Although the fate of most duplicated genes is in fact loss through pseudogenization [1], WGDs provide a massive number of contemporarily created gene duplicates, of which only a small fraction seems to have contributed to some major biological innovations and/or elaborations.

It has become increasingly clear that rather than just the functional divergence of the coding regions and/or regulatory sequences of individual genes, the rewiring of the regulatory network containing these individual components following WGD is of major importance [133,134]. A body of literature exists demonstrating that regulatory and developmental genes in particular are retained in excess after WGDs. This is most likely due to dosage-balance constraints, i.e. selection against loss of individual components of completely duplicated macromolecular complexes and/or pathways, because this would disrupt their overall stoichiometry [135–139]. Retention of balance-sensitive duplicates thus does not provide an immediate evolutionary advantage, but results from the fact that their loss would lead to an immediate disadvantage. In this respect, the retained regulators may be considered an evolutionary spandrel [129,135], which might later on have facilitated the evolutionary innovations and/or diversifications observed in many of these post-WGD lineages [2,3,140]. Selection to maintain dosage balance eventually relaxes over time allowing functional divergence in the context of the environment [138,141], so that part of the duplicated network can be rewired to execute novel functions [133]. However, the underlying mechanisms are currently unclear. Gene duplication has been shown to contribute to innovations even after prolonged periods between the original duplication event and the origin of novelty [142], suggesting that individual components of these duplicated networks can undergo neo- and subfunctionalization in accordance with Ohno's classical models [131,132] even long after the duplication event itself. Some of these processes could have caused network-rewiring events that could help explain the vast post-WGD success observed in some of the plant families that experienced a WGD at the K–Pg boundary.

There are many examples that support the role of network rewiring over time. The ability for anaerobic fermentation in yeast has been associated with global rewiring of its transcriptional network after genome duplication, involving changes in the promoter regions of several genes such as the loss of specific regulatory motifs [143,144]. Similarly, the abundance of teleost fish pigmentation synthesis pathways has been attributed to the teleost WGD through rewiring in combination with subfunctionalization of existing pathways [145]. In plants, the *gamma* hexaploidy at the base of the core eudicots resulted in expansion of MADS-box gene families, key regulators of reproductive development, which through rewiring of their interaction network in combination with neo- and subfunctionalization, acquired roles in several major plant developmental processes [8,146].

## 6. Conclusion

Advances in plant genomics, molecular sequence divergence estimation and high-performance computational solutions allow us to address questions about the role of genome duplication that were previously impossible to investigate. It should be emphasized that the fate of most newly formed polyploids appears an evolutionary dead end through outcompetition by their diploid specialized progenitors [147–149] because of a whole range of associated negative effects such as minority cytotype exclusion [90], severe meiotic and mitotic abnormalities [150] and ploidy-associated genomic instability [79]. Nevertheless, it appears that there exists a strong link between environmental stress and/or fluctuation and genome duplication, as currently supported by both present-day polyploids and palaeopolyploids at the K–Pg boundary. Could unreduced plant gamete production have increased polyploid formation at the K–Pg boundary? Alternatively, can the apparent prevalence of polyploids at the K–Pg boundary be explained by their increased adaptability? Or do we observe the signature of another mechanism and/or pattern that currently remains elusive, perhaps because both dating of such ancient events and making generalizations about current polyploids remain particularly problematic? In any case, this polyploid heritage may afterwards have fuelled evolution of biological innovations and speciation in the context of newly encountered conditions during the Cenozoic, through extensive network rewiring and functional diversification of regulatory and developmental genes that were originally guarded against loss through mechanistic dosage-balance constraints. Polyploids in some sense thus seem reminiscent of the ‘hopeful monsters’ advocated by Goldschmidt [151] (M. Freeling 2009, personal communication), at least at the genomic level, whereas their full potential at the phenotypic level can only be realized given time and the right conditions [52]. It thus appears that the role of the environment in both polyploid establishment and their evolutionary success constitutes an important aspect that merits further investigation.
